# Exploring cross-national public support for the use of enhanced weathering as a land-based carbon dioxide removal strategy

**DOI:** 10.1007/s10584-021-03050-y

**Published:** 2021-03-19

**Authors:** Elspeth Spence, Emily Cox, Nick Pidgeon

**Affiliations:** Understanding Risk Research Group and Leverhulme Centre for Climate Change Mitigation, School of Psychology, Cardiff University, Cardiff, UK

**Keywords:** Public perceptions, Enhanced weathering, Climate engineering, Affect

## Abstract

This study explores how public attitudes across three countries influence support towards terrestrial enhanced weathering, whereby silicate minerals are applied to agricultural land to remove carbon dioxide from the atmosphere. An online survey was administered in Australia (*N* = 1000), the UK (*N* = 1000), and the USA (*N* = 1026) where there are ongoing field trials of this technique. Findings are similar across all three countries with many participants unfamiliar with enhanced weathering and unsure about supporting the use of enhanced weathering. Results show that positive affect is the main predictor for support of this technique, along with perceived benefits and level of concern about climate change. Open-ended questions asking why respondents would or would not support the use of enhanced weathering elicit mainly affective concepts, with enhanced weathering seen by individual respondents as either something mainly positive or mainly negative, with others saying it sounds risky and/or would have impacts on the environment. The way in which enhanced weathering is communicated is likely to influence support of the use of this strategy so must be undertaken carefully. Overall, our findings show that it is imperative to continue to engage the public, thereby allowing their views to be incorporated as enhanced weathering technology develops over time.

**Electronic supplementary material:**

The online version of this article (10.1007/s10584-021-03050-y) contains supplementary material, which is available to authorized users.

## Introduction

Climate change has become a much more salient issue for publics around the world over the past few years with the highest recorded temperatures being set and an increase in events such as heatwaves, flooding, drought, and wildfires. Unless rapid and challenging policy changes are made, it is unlikely that the aspiration of the Paris agreement to limit global average temperature increase to close to 1.5 °C will be achieved (IPCC 2018). In particular, in order to approach a goal of net zero carbon emissions, urgent action across all levels of society is required, action which is likely to include at least some use of Carbon Dioxide Removal (CDR) technologies (National Academy of Sciences, 2019; Ripple et al., 2019; Royal Society and RAEng, 2018). The current study examined public understanding and risk perceptions of terrestrial enhanced weathering (EW), a potential CDR strategy that is currently being researched as part of approaches to reduce climate change. There are several different forms of enhanced weathering, but this study focused on application of silicate minerals (e.g. basalt) onto agricultural land, an approach which has gained considerable scientific and media attention in recent years. Biogeochemical reactions within soil ecosystems transform atmospheric carbon dioxide into carbonates, which become sequestered in soils and the oceans on a long-term scale. Some estimates suggest such terrestrial weathering could indeed, if applied at global scale, contribute significantly towards global CDR goals (Beerling et al., 2020; Kelland et al., 2020). Aside from removing carbon dioxide from the atmosphere, terrestrial enhanced weathering may have co-benefits including slowing ocean acidification, benefitting croplands, and improving soils (Hartmann et al., 2013; Beerling et al., 2018). Research has shown that in controlled environmental settings, adding basalt to crops increased soil potential to store four times as much carbon dioxide and also increased crop yields by up to 20% (Kelland et al. 2020; Beerling et al. 2020). However, there are numerous considerations that are likely to affect the efficiency and feasibility of enhanced weathering as a sequestration technique. For example, there are high energy costs associated with mining, grinding, and spreading minerals, thereby reducing the potential for net CO_2_ removal (Moosdorf et al. 2014), with these processes likely having additional environmental and ecological consequences. There are also a range of ethical and social concerns; most of which are also common to other CDR strategies, including spatial and inter-generational inequalities in the risks associated with deployment. For example, those living where this strategy is likely to be most effective at scale (e.g. in countries in the Global South) are the least responsible for historical emissions (Cox et al., 2018; Lawford-Smith and Currie, 2017).

Many CDR approaches, including terrestrial enhanced weathering, have uncertainties over their environmental impacts, potentially altered ecosystems, financial costs, and the need for land and space to build facilities and store emissions (Lomax et al. 2015). CDR approaches range from established land-use change such as afforestation or peatland restoration through to more novel strategies including bio-energy with carbon capture and storage (BECCS), enhanced weathering, or even direct chemical capture of CO_2_ from the air (Fuss et al. 2016). Many of these technologies are still in the very early stages of development, with enhanced weathering currently undergoing field-scale research trials. For new technologies, recommendations for ‘upstream engagement’ demonstrate that constructive societal dialogue should be carried out while research and development are still in the early phases, with deliberations amongst relevant parties helping to shape technologies, identify concerns, and inform decision-making (Wilsdon and Willis 2004; Rogers-Hayden and Pidgeon 2007; Macnaghten 2020). Currently there is limited public awareness and even less research into public perceptions of CDR approaches and technologies. It is clear that if these technologies are ever to be deployed at scale, a range of ethical, legal and moral issues will first need resolution, and many of these issues will impact upon societies and publics (Corner and Pidgeon 2010; Cox et al. 2018). Accordingly, inclusion of public voices in debates about CDR is essential to determine preferences for these emerging technologies, to assess what might influence public acceptability and support, and to ensure that any innovation pathways and governance systems adopted are responsive to societal concerns (Parkhill et al., 2013; Pidgeon, 1998; Rayner et al., 2013).

The CDR strategies described above are commonly defined as a sub-set of ‘geoengineering’, which refers to deliberate large-scale manipulation of the Earth’s climate that could either be through solar radiation management (SRM) or carbon dioxide removal (CDR) (Royal Society, 2009). SRM technologies involve reflecting sunlight back into space away from Earth and are considered particularly controversial because of their significant uncertainties and possible environmental consequences, as well as the fact that they do not directly reduce CO_2_ (Bellamy et al. 2013; Pidgeon et al. 2013). There is not one broad public perception towards climate engineering but a range of possible responses depending on the specific technology type, its governance arrangements, and associated risks and challenges which will vary for each. It is important that these strategies should be considered separately, and broad categorisations avoided, such that public discourse and governance can be made more useful and relevant to future decisions about specific strategies (Colvin et al. 2020). As research into many of the more ‘upstream’ approaches is still in its initial stages, it is necessary to ascertain what shapes public perceptions of climate engineering and emerging technologies to help predict what could help inform public understandings of individual technologies and proposals.

### Public perceptions

Early work into public perceptions of climate engineering has shown generally low levels of awareness, with some evidence that those who are more concerned about climate change are more supportive of climate engineering (Pidgeon et al., 2012). In addition there is commonly a preference for CDR methods over SRM (Pidgeon et al., 2012; Scheer and Renn, 2014). Some of the previous literature has focused specifically on SRM, generally showing low levels of acceptance and support (Mercer et al. 2011; Macnaghten and Szerszynski 2013; Visschers et al. 2017), with risks generally perceived to be greater than benefits (Sütterlin and Siegrist 2017).

As the debate about the potential need for climate engineering becomes more intense, there is an increase in work exploring what predicts public support for specific types of strategy across both SRM and CDR. Corner et al. (2013) demonstrate how climate engineering evokes a complex set of ‘messing with nature’ narratives. In particular, people often view it as simply interfering with natural processes and ultimately not dealing with the main cause of climate change: anthropogenic carbon emissions. Wolske et al. (2019) have also explored the extent to which specific CDR strategies are perceived to be tampering with nature, finding greater support for afforestation and reforestation (AR) over BECCS and DAC (direct air capture), with AR perceived to mess with nature to a lesser extent. This effect was stronger in participants who were against human intervention in natural processes. This preference for AR is common, in part because it is perceived to be a more ‘natural’ approach as compared to other more technological strategies (Corner and Pidgeon 2015; Campbell-Arvai et al. 2017).

One common methodological consideration across much of this risk perceptions work is the need to provide study participants with information about each technique due to low levels of familiarity, which may include giving information about potential risks and/or benefits. Usually this information is made as neutral and balanced as possible to avoid framing effects. When awareness is low, however, information provision is likely to influence level of support as both perceived risks and benefits contribute to attitude formation, with those perceiving greater risks than benefits tending to be more opposed to new technologies (Slovic 2000). There is evidence that providing further information about both the risks and benefits of different climate engineering strategies, as opposed to merely providing a description of techniques, tends to result in reduced support (Braun et al. 2017; Wolske et al. 2019), and this has been attributed to how serious respondents perceived climate change to be, or alternatively the extent to which a particular strategy is thought to be tampering with nature. In the Braun et al. study, those who saw climate change as more serious were more accepting of measures but perceived the information more negatively than those who did not perceive climate change as a serious issue. Wolske et al. (2019) likewise found that after respondents had read further information about three CDR strategies, BECCS was seen as most likely to tamper with nature and therefore had reduced levels of support.

Another important component of risk perception is the role of affect: the positive or negative feeling instinctively associated with a risk issue or technology (Slovic et al. 2007). For example, Merk and Pönitzsch (2017) clearly showed that those who felt more positive about stratospheric aerosol injection (one type of SRM) had an increased benefit perception and decreased risk perception, and the reverse for those with negative affect. The affect heuristic allows people to evaluate novel technologies with limited to no knowledge about the topic. Sütterlin and Siegrist (2017) show that even with limited information, people evaluate SRM negatively, with this producing a stronger negative affective response in those given further information about possible risks. The authors surmise that people no longer only have their affective evaluation of climate change to rely on but can now utilise the information received about SRM, which means how technology is described will likely predict the relation between climate change concern and perceptions of climate engineering.

### Public perceptions of enhanced terrestrial weathering

Most recent public perceptions literature has focussed on a range of climate engineering strategies, across both CDR and SRM, allowing for comparisons between specific strategies. However, there are very few survey studies that have included enhanced weathering, which is the key focus of this research. Wright et al. (2014) did include enhanced weathering in a survey investigating perceptions of six climate engineering strategies (three CDR and three SRM) in New Zealand and Australia. There were higher net positive associations made with the CDR strategies, all being judged as more controllable and sustainable than SRM. This was also true for enhanced weathering, although in this study it was not viewed as a very distinct concept by respondents: in other words, it did not produce particularly positive or negative associations when considered by people, in comparison to the other climate engineering strategies judged. Follow-up work included a further two countries (the UK and USA) replicating this finding, but also explored support for small-scale field trials (Carlisle et al. 2020). Results illustrated mixed support for trials with a similar pattern across all four countries consistent with earlier studies. CDR was more supported than SRM. Participants also displayed considerable ambivalence around supporting trials for all strategies, with many neither agreeing nor disagreeing that trials should occur, including in the case of enhanced weathering. Jobin and Siegrist (2020) also recently explored the key drivers of public support for 10 different climate engineering strategies (seven CDR and three SRM) finding highest support for afforestation and lowest support for SRM. Specific to enhanced weathering (alongside other soil-based CDR strategies), there were low levels of public knowledge. With regard to general support, higher perceived benefits increased support for use of enhanced weathering and higher perceived risks (and perceptions of tampering with nature) decreased support for deployment.

The studies to date provide an indication of how the public perceive different CDR strategies compared to each other, including the few studies described above incorporating enhanced weathering. However, building understanding of risk perceptions of any single CDR technology also requires investigating specific approaches in greater detail. This more targeted approach allows for greater provision of technology-specific information and survey questions and scales which are tailored to the technology under consideration. Accordingly, the present study examined perceptions towards enhanced weathering as one possible CDR strategy to help mitigate climate change, building on previous survey work with respondents from the UK (Pidgeon and Spence, 2017). This earlier work, utilising a limited range of technology specific items, confirmed that, as with other climate engineering approaches, enhanced weathering was unfamiliar to most respondents, while those who were more supportive of the approach also felt greater positive affect and perceived more benefits than risks of the approach.

### The current cross-national study

To extend previous work, we surveyed the populations of three Westernised countries where small-scale field trials of the enhanced weathering techniques described previously are already occurring (the UK, the USA, and Australia). The survey examined what influences support for deployment of enhanced weathering and whether this differs between countries, and the extent to which support is related to people’s beliefs about climate change. All three countries have seen significant increases in climate concern in recent years. In Australia, following several extreme heat and wildfire seasons over recent summers, the percentage of respondents very concerned about climate change increased to 47% in January 2020 (TAI 2020), a percentage which has almost doubled since 2010 (Reser et al. 2012). In the UK, 40% expressed being ‘very’ or ‘extremely’ worried in late 2019, a figure which had doubled since 2016 (Steentjes et al. 2020). In the USA, there had likewise been an increase in those most worried about climate change with 31% in 2019 classified as ‘alarmed’, a segment of the US population that had tripled between 2014 and 2019 (Leiserowitz et al., 2020). Accordingly, this is the first study to explore public perceptions of enhanced weathering in detail across these three countries in light of increases in climate concern, and will help provide initial insights into how people rationalise their support for such a novel CO_2_ removal approach.

We hypothesised that people would be unfamiliar with enhanced weathering, as it is a novel approach to CO_2_ removal (Wright et al. 2014; Carlisle et al. 2020), while both positive affect and concern about climate change would be positively related to support for enhanced weathering (Pidgeon and Spence, 2017), and that those who perceived more benefits would also be more supportive (Merk and Pönitzsch 2017). We also included an option eliciting open-ended responses to help explain why people would or would not support development of enhanced weathering, as here we expected a high degree of ambivalence (Carlisle et al., 2020; Pidgeon and Spence, 2017).

## Method

### Participants

A broadly nationally representative sample from each country was recruited through Qualtrics using panel databases with respondents aged 18+ in terms of age, gender, education, and geographical region. The survey was completed online in the USA (*N* = 1026), the UK (*N* = 1000), and in Australia (*N* = 1000) during February and March 2019. The US sample was slightly more highly educated than the US population, with more achieving a postgraduate degree. In contrast, the Australian sample overrepresented those with a lower level of education as compared to the general population. For a breakdown of the demographics, please see ESM1.

### Procedure and materials

This survey was designed to measure perceptions of climate change as well as enhanced weathering to explore support for this carbon dioxide removal strategy. See ESM2 for all items reported. The survey was carried out alongside a mixed methods study into public perceptions of CDR in the UK and USA, reported in Cox et al. (2020), which used qualitative deliberative workshops along with results from a separate section of this survey exploring perceptions of CDR.^1^

### Climate change perceptions

The survey asked a series of questions about climate change, designed to elicit general attitudes towards climate change. Respondents indicated how concerned they were about climate change in response to the question ‘How concerned, if at all, are you about climate change?’ (4 = very concerned, 1 = not at all concerned, with ‘do not know’ and ‘no opinion’ options also provided) (Steentjes et al. 2017). In a second concern item, we also asked about worry about climate change: ‘How worried are you about climate change? (five-point scale ranging from ‘not at all worried’ to ‘extremely worried’) (Chryst et al. 2018). Respondents also indicated the extent to which they agreed or disagreed with the following statement (five-point scale as well as provision of a ‘do not know’ option): ‘To what extent do you agree with the following statement about science: Science and technology will eventually solve our problems with climate change’ (Steentjes et al. 2017).

### EW perceptions

**Fig. 1:**
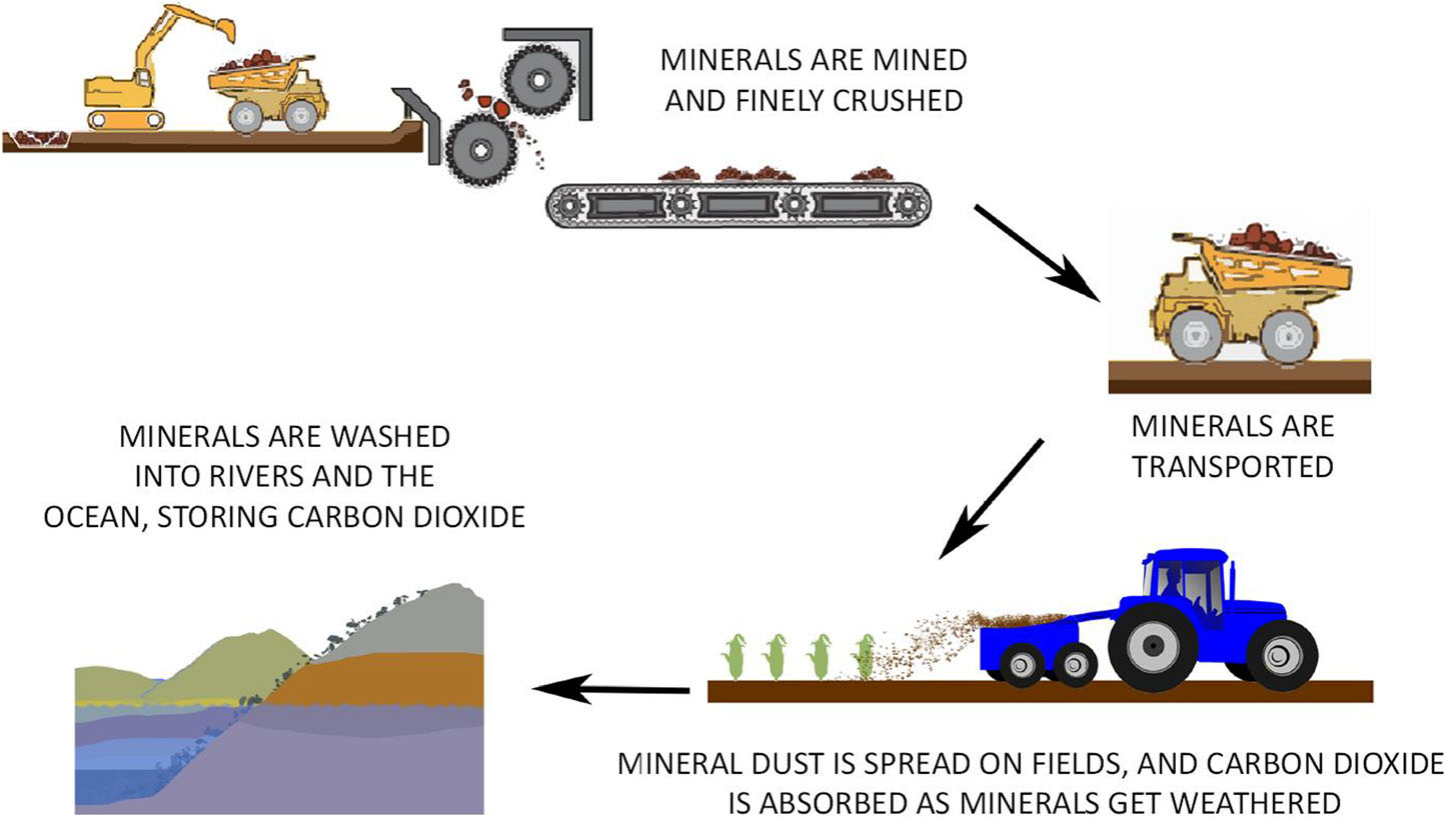
Information about enhanced weathering shown to respondents. “Weathering is the breakdown of rocks and minerals through rainwater, extreme temperatures, and living organisms removing carbon dioxide from the atmosphere over thousands of years. Enhanced weathering speeds this process up by mining and crushing minerals that absorb carbon dioxide, to increase the surface area exposed to the atmosphere. These crushed minerals are then spread on agricultural fields which could improve growing conditions for crops. The carbon dioxide eventually ends up dissolved in water or as a mineral on the ocean floor. A large amount of energy is needed for mining, crushing, transporting, and spreading the minerals and may harm the diversity of animals and plant species in the environment”

Respondents received a short description including a visual graphic showing the process of enhanced weathering (see Fig. 1), before being asked to share their initial ‘top of mind’ associations with the question, ‘Please tell us what thoughts or images came to mind when reading this information about enhanced weathering?’ The stimulus materials deliberately avoided framing the processes of enhanced weathering as a ‘natural’ approach (see Bellamy and Lezaun 2017; Pidgeon 2020) as it involves speeding up rock weathering that typically takes thousands of years. The material also described the full process from initial mining of material through to CO_2_ sequestration.

We also wanted to determine to what extent the respondents felt that enhanced weathering was risky or beneficial on a range of specific potential risks or benefits. These included four risk items such as ‘Mining and crushing minerals for enhanced weathering could cause environmental damage’ with the four items creating a reliable risk perception scale (this scale showed good internal reliability as *α* = 0.81). To measure benefit perceptions, four benefit items such as ‘Enhanced weathering could improve growing conditions for crops’ were used, with the scale also showing good reliability as *α* = 0.86 (ESM2). Responses were on a five-point scale from ‘strongly agree’ to ‘strongly disagree’. Respondents were also asked about their affective response to enhanced weathering: ‘In general, how do you feel about enhanced weathering’ with responses on a five-point scale ranging from ‘very negatively’ to ‘very positively’. In addition, respondents were asked to what extent they agreed or disagreed with four statements measuring the idea that enhanced weathering may be ‘messing with nature’ taken from Visschers et al. (2017) including ‘enhanced weathering disturbs the order of nature’ (messing with nature scale, *α* = 0.83). It is important to note that our choice of statistical methodology, while widely used in most similar work on risk perceptions, inevitably creates some analytic limitations. Notably, there is a methodological debate around the use of parametric analyses such as regressions, which are widely used when examining ordinal data such as the Likert scales employed here), with this approach being criticised by some for its suitability (Liddell and Kruschke 2018). Parametric statistics are, however, more readily interpretable and for most practical purposes (e.g. unless the underlying variables are heavily skewed) parametric tests yield robust findings (Norman 2010).

Finally, respondents were asked about their support for the use of enhanced weathering across three different items at various points within the survey including ‘would you support the use of enhanced weathering as a way to reduce carbon emissions’ with a five-point scale ranging from ‘not at all’ to ‘definitely’ (support for use of enhanced weathering scale, *α* = 0.84). They were also asked to indicate reasons for their support or not for this strategy in an open-ended item. To examine these open-ended items, an inductive thematic analysis was carried out and a coding frame developed (Braun and Clarke 2006). Analysis was data-driven with themes initially identified based on the open-ended responses without pre-determined notions of relevant concepts to the study. These were refined over time incorporating more theoretically appropriate themes that reflected the public perceptions and climate engineering literature more closely. Two independent coders were used to ensure the transparency of the framework and that agreement was satisfactory. Cohen’s kappa (measuring inter-rater reliability) was 0.74 showing there was a high rate of inter-rater agreement. Differences for responses where agreement was not initially met were discussed and resolved between the two coders. More details can be found in ESM3.

## Results

In this section, we outline results across both the quantitative survey responses and the qualitative response answers. Section 3.1 explores responses to the quantitative climate change questions. This is followed by Section 3.2, which describes key enhanced weathering themes in turn based on both qualitative and quantitative results: familiarity, associations, risk and benefit perceptions, affect, and support for enhanced weathering.

### Climate change

The majority of respondents across all three countries were very or fairly concerned about climate change with levels between 72 and 81%, with those in the UK most concerned. In this regard, the fieldwork occurred during a period in which the group Extinction Rebellion had been intensifying its UK campaigning to highlight the climate emergency, while in Australia drought and large bushfires and in California two of the largest wildfires on record had all occurred within the previous 6 months. Fieldwork was also conducted before the upheaval caused by Covid-19. We also found 39–41% very or extremely worried with a further 33–40% stating that they were somewhat worried, in line with other recent findings using this item in the UK (Steentjes et al. 2020).

We asked about the belief that science and technology could eventually solve climate change. More people in Australia disagreed with this statement (34%) than agreed (28%). The US and UK respondents were slightly more optimistic that science and technology would solve our problems with climate change, with 32% in the USA agreeing and 30% disagreeing, and 30% in the UK agreeing and 27% disagreeing. However, many people neither agreed nor disagreed with this statement (34% in the USA and Australia, and 37% in the UK).

### Enhanced weathering

Across all three countries, it was clear that enhanced weathering was unfamiliar to respondents, with a majority in all three countries stating that they had never heard of enhanced weathering before (74% in Australia, 70% in the UK, and 62% in the USA).

We followed up by including an open-ended item to determine what initial associations came to mind for respondents when they heard the words ‘enhanced weathering’. Within the three countries, there were few differences in the ‘top of the mind’ associations. When analysing the responses for this item, we excluded responses that were left blank, responses that did not make any sense or were unrelated to the question, and responses stating that climate change was not real or a valid issue, on the grounds that these responses failed to answer the question (12.7% of the sample). Firstly, we found that around a fifth of people stated that they did not know about enhanced weathering or found it too confusing or complicated to understand. Second, a large proportion of initial responses mentioned affect and emotion, with many saying that enhanced weathering was either positive or negative. For example, many said that it sounded like a good idea or was interesting, while others said that it sounded bad or was of concern to them. Some went further stating that enhanced weathering sounded scary or dangerous. We also found that many felt that enhanced weathering was risky and would cause more damage or problems, with some referring to it as a temporary solution or a quick fix. This response captured all of these initial thoughts: “I thought this was a very clever solution but worried about the impact on animals and plants and if it will just cause other problems”*,* seeing enhanced weathering as a positive idea but also concerned about its impacts and knock-on effects. Another spontaneous association was the concept of naturalness, with comments indicating whether enhanced weathering was a natural or unnatural process, that it was human-made, or that nature could in fact fix itself.

There were other associations that were more descriptive and related to the content of the information that respondents had read. One of the most common responses from people in all countries was related to concerns around possible impacts on the environment. This included damage to the oceans, harm to organisms, and impacts on waterways and the terrestrial environment: *“*That doesn’t sound good because it’s going in the water. The water is vital and important for us and the environment”*.* In contrast, impacts on humans and health were rarely mentioned by people and mainly related to food, water supply, and dust causing health issues. People also mentioned costs including both environmental and financial costs, with industry seen as possibly taking advantage: “it sounds like profit for the mining industry and a significant cost to the environment”. This also included mentions of resources required to carry out enhanced weathering. Other associations linked to the perception of mining as being industrial and again being a resource-intensive process, with responses also concerned about the use of minerals as a resource and how effective or polluting minerals may be during initial mining, transportation, and application to fields.

For the questions relating to specific risks and benefits, we found that potential risks of enhanced weathering gained higher agreement than the possible benefits in all three countries. There was strongest agreement with the item stating that there may be impacts on organisms in the marine environment (74–79%), in line with the results from the deliberative workshops reported in Cox et al. (2020). Respondents were less certain about the benefits of enhanced weathering, with less than half agreeing with the benefit statements. For example, only between 37 and 39% agreed that it may help reduce the impacts of climate change on the ocean. A larger proportion across all respondents were more unsure about the benefits of enhanced weathering than they were about the potential risks.

We wanted to know if enhanced weathering induced positive or negative feelings. On this single item, we found that across all three countries, around half of the sample did not feel either positively or negatively about enhanced weathering with the remaining responses showing little difference between the proportion saying it made them feel negative and those who said that it made them feel positive. There was also agreement that enhanced weathering was indeed ‘messing with nature’, with similar responses across all three countries (62% agreement in Australia and the UK; 57% agreement in the USA).

The support measure for the use of enhanced weathering was explored in multiple regression analyses to assess what background variables influenced this (Jobin and Siegrist, 2020; Merk and Pönitzsch, 2017; Pidgeon and Spence, 2017). The coefficients are reported in Table 1.

**Table 1:** Regression analysis showing predictors that influenced support for use of enhanced weathering across Australia, the UK, and the USA

Predictor variables	AUS	UK	USA
	*β*	*β*	*β*
Gender (male)	.01	.03	.03
Education (university)	−.01	.01	.01
Age	−.02	.01	−.06**
Concern CC	.08***	.09***	.12***
Science/Tech solve CC	.06**	.06**	.07**
RisksEW	−.11***	−.10***	−.12***
BenefitsEW	.34***	.36***	.36***
Messing with nature	−.13***	−.12***	−.07***
Affect (positive)	.43***	.43***	.42***
R^2^ adjusted	.69	.58	.66

A multiple regression was carried out for each country. *β* shows the standardised coefficient**.** R^2^ adjusted shows variance explained in the overall model

Significance: *p < .05, **p < .01, *** *p* < .001

A consistent regression pattern was found in all three countries, although the model was slightly less predictive in the UK sample (58% of variance explained) as opposed to Australia (69%) and the USA (66%). The pattern of coefficient values was very similar across countries for each variable included, indicating a similar degree of relative influence of each predictor variable, and with the larger coefficients in Table 1 showing which predictor variables provided the most overall influence on support. Table 1 shows that affect is the strongest predictor in determining support for the use of enhanced weathering across all three countries; that is, the more positive people feel about enhanced weathering, the greater their support for it (e.g. in the US model, *β* = 0.42 *p* < .001). Perceived benefits (positive) and perceived risks (negative) were also related to support in the directions predicted, with benefits proving relatively more important; the coefficient values were three times as strong for perceived benefits than they were for perceived risks (e.g. in the US model *β* = .36 and *β* = −.12, respectively). As expected, there was a negative correlation between support and the degree to which a respondent felt that the technique was ‘messing with nature’ although here the coefficient in the USA (*β* = −.07, *p* < .001) indicates a slightly weaker relationship than in the UK (*β* = −.12, *p* < .001) or Australia (β = −.13, *p* < .001). Also as predicted, concern about climate change was positively related to support in all three countries, as was a belief that science and technology would eventually solve climate change.

Across the three countries, most said that they were unsure about supporting enhanced weathering (42.8% in Australia, 45% in the USA, and 49.7% in the UK), with around a third of people saying that they would support enhanced weathering (33.9–35.7%) and a fifth or less saying that they would not support it (15–21.5%). As well as measuring whether respondents would support the use of enhanced weathering, we asked people to provide their rationale for their answer in an open-ended item about why they would or would not support enhanced weathering; see Table 2 for the most common responses. Analysis showed that across all three countries, people voiced similar reasons and concerns behind their decisions.

**Table 2:** Most common responses in each country shown by whether they did or did not support enhanced weathering or were unsure either way

Response category	Summary
Yes (would support enhanced weathering)	
Positive	Generally positive comments that do not refer to the environment or humans
Benefits	Responses that mention it being beneficial or benefit outweighing the risk
Positive for environment	Comments around the environment improving or benefiting the planet
None/do not know	Left blank, none, cannot think of any, need to know more
No (would not support enhanced weathering)	
Risks	Any mention of it being risky or harmful or not knowing about damage yet, or being more risky than beneficial
Impact environment	Responses that mention damage to environment, animals and wildlife and concern about environmental damage
Band-aid/alternative	Responses that talk about it being a band-aid, or temporary fix
None/do not know	Left blank, none, cannot think of any, need to know more
Unsure (about supporting enhanced weathering)	
None/do not know	Left blank, none, cannot think of any, need to know more
Research/evidence	Any mention of research, evidence, need for trials or testing, it not being proven or having proof
Risks vs benefits	All responses that seem unsure about cost/benefits of process or a desire to see further information to make a decision based on risks/benefits

Broadly, it is clear that those supportive of enhanced weathering saw it as a positive thing or that it was likely to be beneficial overall. Another common response was that it would improve the environment or help with climate change and reduce damage: “We need to get the carbon out of the atmosphere, just cutting down on emissions will not reverse the damage that has already been done. We need a range of techniques to trap carbon”*.* There were also a proportion of respondents who could not think of a reason for why they supported enhanced weathering (from 8.8% of supporters in the USA to 18% in the UK), although these numbers are small when considering that only a third of the overall sample supported the use of enhanced weathering.

For those who said that they would not support enhanced weathering, the most common reason was the risks that it could bring, with concerns about it causing more harm or damage and not being worthwhile compared to possible benefits. Many were also worried about damage to the environment including to animals and wildlife across all three countries. Lastly, others felt that enhanced weathering was a temporary fix or a ‘band-aid’ which did not reduce emissions, with others proposing alternative solutions which did: “I don’t see this as a way to reducing emissions, only a short term solution to deal with existing levels and the potential long term negative effects are a waste of time and effort that could be spent actually addressing the real problem”*.*

There were many who were simply not sure about whether they would support enhanced weathering or not, with many simply stating they did not know. Others mentioned the need for more research to be conducted and for more evidence that it was a viable proposition, with some discussing the uncertainty between the balance of risks and benefits of enhanced weathering: “I feel there is not enough evidence either way yet so there needs to be experiments done before I would stand on either side of the argument for enhanced weathering”.

## Discussion

This research is the first comparative international study to focus solely on perceptions of and support for enhanced weathering as a carbon dioxide removal option in Australia, the UK, and the USA, three Westernised countries where small-scale field trials of this strategy are currently underway. The study found positive affect to be the main driver of support along with perceived benefits, with those more positive about enhanced weathering also more supportive of its deployment. These findings were broadly similar across countries, with awareness of the technology also remaining very low in all three (cf. Carlisle et al., 2020; Pidgeon and Spence, 2017). Despite some very clear political and cultural differences between the countries and their very different national stances on climate change policy at the time at which the survey was conducted, our findings also suggest that the idea of enhanced weathering evokes a common initial ‘mental model’ (Morgan et al. 2002) in members of the public in these three anglophone countries at least. A ‘mental model’ refers to the idea that people will use their existing beliefs, and any other knowledge and associations that they can access, to create an image or ‘cognitive template’ in their mind of the particular risk issue, its causes, and consequences (Bruine de Bruin and Bostrom 2013). Understanding how people conceptualise enhanced weathering in this way will help identify what might influence public support for this technology in future.

With a topic which is so unfamiliar to people, establishing top of the mind associations is a commonly used elicitation technique to determine initial responses towards an environmental or technological issue (Tvinnereim and Fløttum 2015). The concepts elicited here were mainly affective, with enhanced weathering seen by individual respondents as either something mainly positive or mainly negative, with others saying it sounded risky and/or would have impacts on the environment. When knowledge is low, the affective system is often highly influential in public evaluations, as seen across empirical work on both geoengineering and other unfamiliar technologies (e.g. Merk and Pönitzsch, 2017; Poortinga and Pidgeon, 2006; Satterfield et al., 2009). In this respect, the regression analysis confirmed that affect was, as expected, the strongest independent predictor of support for the approach. The results also demonstrate that generally more risks than benefits were perceived, and respondents across all countries were especially concerned about environmental impacts. These findings suggest that, if deployment at scale is ever to be achieved in a societally robust manner, enhanced weathering must be carried out and governed in an environmentally benign way which simultaneously maximises co-benefits (Smith et al. 2019) while minimising scientific uncertainties around environmental impacts, particularly in the oceans (also Cox et al. 2020, 2021).

An important consideration for this study was the framing of information presented to participants, a difficult issue to resolve when awareness is so low. Although we wanted to make this as balanced as possible, providing both a description of the technique and possible outcomes may well have influenced the results (see Braun et al., 2017). Wolske et al. (2019) suggest that support for CDR strategies may be reduced depending upon the types of risks and benefits described, although in their study they found that this varied both by CDR strategy and the extent to which a strategy was perceived to ‘tamper with nature’. In common with this and other research, the current study also found that those who saw enhanced weathering as something that would ‘mess with nature’ were less supportive of the approach if it were to be deployed (Corner et al. 2013; Merk et al. 2015; Visschers et al. 2017; Wolske et al. 2019). In future, it would be worthwhile to explore path relationships between important factors to determine how support for enhanced weathering is influenced in a more detailed way. For example, it would be useful to know if those who perceive this approach very positively automatically presume there are greater benefits, or if those who see enhanced weathering as something that will mess with nature also see it as high risk. By exploring pathways between important factors, a more nuanced view into opinion formation towards enhanced weathering and other CDR approaches can be developed (Cummings and Rosenthal 2018).

Consideration of such framing effects sets an important future research topic, alongside how various publics perceive the severity of climate change. In particular, it would be a useful extension of these studies to explore how support for CDR strategies is influenced explicitly by information frames, including whether information about risks, benefits, or degree of ‘naturalness’ is provided (Corner and Pidgeon, 2015; Jobin and Siegrist, 2020). Recent deliberative work with publics in both the USA and UK has suggested that framing enhanced weathering as a soil amendment, rather than a climate change technique, might result in different perceptions, particularly amongst those who perceive a fundamental contradiction between the heavy machinery required for enhanced weathering and their desired ‘vision’ for a sustainable future society (Cox et al., 2020). It might also be worthwhile exploring public responses to non-climate-based framings of enhanced weathering, in particular agricultural framings (e.g. impact on crop yield), and as an intervention to counter local ocean acidification. However, it should not be naively assumed here that different information frames might yield a route to achieving greater public acceptance of CO_2_ removal in the face of accelerating climate change. Attempts to overly frame the risks, benefits, and other perceived characteristics of a technology during its initial stages in the search for ‘societal acceptance’ are at best ethically problematic, while also risking significant amplification of risk perceptions and a loss of trust in the science of CDR if major impacts with one of these technologies were subsequently to emerge (Leiss 2003).

We also saw that those who were more concerned about climate change were more supportive of enhanced weathering, reinforcing findings from other recent research (Braun et al., 2017; Pidgeon and Spence, 2017; Jobin and Siegrist, 2020). This is an important result, because although we might assume that those with higher concern for climate change would automatically support low- or negative -carbon technologies, previous research demonstrates that this is not always the case. For example, nuclear power has a carbon footprint well below that of electricity generated by coal or natural gas, yet higher climate change concern invokes more negative perceptions even when controlling for a range of demographic variables (Corner et al., 2012; Sonnberger et al. in press). Some studies of Carbon Capture and Storage (CCS), itself a key component of some CDR proposals, also find an inverse relationship between support for the technology and climate change concern (Terwel et al. 2012; Kraeusel and Möst 2012), although findings across a range of CCS studies provide mixed evidence on this point (L′Orange Seigo et al. 2014). Therefore, as research and development into new technologies or expansions of known technologies continue, caution should be applied; discourses of climate emergency have been a key recent public concern in many countries, but simply presenting a technology as having potential to help with this does not necessarily reduce other aspects of public risk perceptions. It is important to emphasise here that acceptance of enhanced weathering, as with many other CDR approaches, will at best be conditional, and our deliberative work conducted in the USA and UK confirms this, with perceptions and support contingent upon the way in which enhanced weathering will be carried out, particularly regarding issues of environmental impacts, scale, mining, and the transport of rock materials (Cox et al., 2020).

It is now more than 4 years since the Paris Climate Agreement came into force, with the potential use of large-scale CDR approaches increasingly being discussed in the policy sphere as one potential route for approaching the target that Paris requires (Scott and Geden 2018). However, low public awareness of CDR persists, and will provide a clear communication challenge in the climate science and policy communities (Cummings et al. 2017). Our findings also suggest that there is an urgent need to engage a wide set of publics about enhanced weathering and its potential contribution to meeting national climate targets. As technologies are developed (and in some places scaled-up over time), such engagement with publics and communities will also need to form a part of the wider global debate about making progress towards a net-zero world, while giving detailed consideration also to localised implications (risks and benefits) in places where significant deployment might occur. In this survey, open-ended responses rationalised people’s support (or lack thereof) for enhanced weathering, identifying valid concerns about possible risks as well as hopes that it could be beneficial and help reduce climate change. Future work could usefully explore how such research into public views can be utilised as a form of ‘social intelligence’ within a process of Responsible Research and Innovation (RRI), in order to steer development of carbon dioxide removal technologies in socially responsible ways (Macnaghten, 2020; Stilgoe, 2015; Stilgoe et al., 2013). In this regard, Low and Buck (2020) point out that, despite the existence for over 10 years of the first clear RRI guidelines for geoengineering in the form of the Oxford Principles (Rayner et al., 2013), there have been no genuine documented exemplars since the stage-gate process which evaluated the social, ethical, and safety aspects of the ‘SPICE’ SRM test-bed project in the UK (see Pidgeon et al., 2013; Stilgoe, 2015). Low and Buck further argue that in any such future effort, RRI researchers, alongside CO_2_ removal scientists and engineers, will have to adopt a fully critical and reflexive approach to any technology-society appraisal processes.

Finally, this current study has elicited similar risk perceptions across three Westernised countries, all with small ongoing field trials but the potential for at-scale deployment. Emerging work has already shown differences when climate engineering has been examined with publics in non-Westernised countries (Visschers et al. 2017; Sugiyama et al. 2020). On the one hand, communities in the Global South with major agricultural systems and where climate is more favourable to the enhanced weathering process (Edwards et al. 2017) may also be more vulnerable to climate change and already suffering the impacts (Althor et al. 2016), hence more supportive due to higher levels of concern about climate change (Carr and Yung 2018). Equally, such communities might consider climate engineering as the imposition of a ‘solution’ favouring the Global North, and for a problem that they have had little historical role in creating (Winickoff et al. 2015). Accordingly, future research must explore how a range of communities, across both Global South and North, perceive the potential risks, benefits, and governance implications of enhanced weathering, taking into account cultural, social, and political influences.

## Supplementary information

ESM 1 (PDF 341 kb)

## Data Availability

The datasets analysed during the current study are available in Cardiff University Open Data Repository: 10.17035/d.2021.0129331305.

## Footnotes

1 The survey questions reported in Cox et al. (2020) elicited attitudes towards CDR, after the climate change items but before the enhanced weathering description and questions (ESM2).
