# Supplementary material for: Exploring cross-national public support for the use of enhanced weathering as a land-based carbon dioxide removal strategy
Source: Clim Change. 2021 Mar 19;165(1-2):23. doi: 10.1007/s10584-021-03050-y (PMC7978169; doi:10.1007/s10584-021-03050-y)
Supplement: Supplementary file 1 — (PDF 341 kb) [file 10584_2021_3050_MOESM1_ESM.pdf]

Electronic Supplementary Material

Exploring cross-national public support for the use of enhanced weathering

Elspeth Spence*, Emily Cox, Nick Pidgeon

*Corresponding author, Understanding Risk Research Group, School of Psychology, Cardiff University, and Leverhulme Centre for Climate Change Mitigation, University of Sheffield, spencee@cardiff.ac.uk

ESM 1 – Demographics for each country: Provides demographic breakdown for each country (Australia, UK and US) compared to census data

ESM 2 – Items from the survey: Items included in the survey in order of presentation to participants

ESM 3 – Open-ended coding frameworks: Full frameworks for coding of open-ended items for initial associations with enhanced weathering and support for enhanced weathering

ESM 4 – Descriptive statistics: Means and SDs for items included in survey across each country

ESM1: Demographics

Demographics include gender, age and education, however we did not collect details on indigenous peoples across the relevant countries. We did include an item in the Australian survey allowing respondents to indicate if they were Aboriginal or Torres Strait. Our sample was over-represented at 3.1% as census data indicates they form 2.5% of the Australian population.

Australian Sample

Australia	Sample (N = 1000)	Australian Census[1]
**Gender**	**%**	**%**
Male	49	49
Female	51	51
**Age (Years)***		
18 – 24	10	10
25 – 34	18	18
35 - 44	17	17
45 – 54	17	17
55 - 64	15	15
65+	23	23
**Education**		
Less/Not stated	43	17
Certificate I - IV	19	31
Advanced Dip/Dip	10	15
Bachelor	19	25
Grad Dip/Grad Cert	3	3
PG Degree	7	8

UK Sample

UK	Sample (N = 1000)	UK Census[2]
**Gender**	**%**	**%**
Male	49	49
Female	51	51
**Age (Years)***		
18 – 24	13	12
25 – 34	17	17
35 - 44	16	18
45 – 54	17	18
55 - 64	16	15
65+	21	21
**Education**		
No qualification	22	23
Other	8	8
Level 1	14	14
Level 2	15	15
Level 3	12	12
Level 4	28	27

[1] Australian Bureau of Statistics (2021). 2016 Census of Population and Housing, https://www.abs.gov.au/census
[2] Office for National Statistics; National Records of Scotland; Northern Ireland Statistics and Research Agency (2016): 2011 Census aggregate data. UK Data Service (Edition: June 2016). DOI: http://dx.doi.org/10.5257/census/aggregate-2011-1

US Sample

US	Sample (N = 1026)	US Census[3]
**Gender**	**%**	**%**
Male	49	49
Female	51	51
**Age (Years)***		
18 – 24	13	14
25 – 34	17	18
35 - 44	16	17
45 – 54	17	17
55 - 64	16	17
65+	22	19
**Education**		
Less than high school	11	11
High School Diploma	29	29
Associate Degree	28	28
Bachelor Degree	17	20
PG Degree	15	11

***Census data for age has been adjusted for population 18+**

[3] US Census Bureau (2021) 2018 American Community Survey 5-Year Estimates, https://www.census.gov/programs-surveys/acs

ESM 2: Items from the survey

Items	Response Scale
Q1. How concerned, if at all, are you about climate change?	Very concerned – not at all concerned, don't know, no opinion
Q2. How worried are you about climate change?	Not very worried – very worried
Q3. To what extent do you agree with the following statement about science:<br>Science and technology will eventually solve our problem with climate change	Strongly agree, Tend to agree, Neither agree nor disagree, Tend to disagree, Strongly disagree,<br>Don't know
Participants were then provided with a short description of 'Carbon Dioxide Removal' (below) and asked questions gauging perceptions of CDR (Q4-5). This data and its analysis was reported in a separate study by Cox et al. (2020)	
Please read the following information carefully:<br>"Scientists and policymakers have become more interested in carbon dioxide removal or 'CDR' as a strategy that may slow or reverse climate change. These strategies remove excess carbon dioxide (CO2) from the atmosphere through various biological, chemical or physical processes. The carbon dioxide would be stored by plants, in soils, or deep underground and in the deep ocean so that it cannot contribute to an increase in the Earth's temperature."	
Q4. Before today, how much if anything, would you say that you know about carbon dioxide removal technologies?	- I know a great deal about carbon dioxide removal technologies<br>- I know a fair amount about carbon dioxide removal technologies<br>- I know just a little about carbon dioxide removal technologies<br>- I have heard of carbon dioxide removal technologies but know almost nothing about it<br>- I have not heard of carbon dioxide removal technologies before today
Q5. Some people believe that carbon dioxide removal technologies may have associated risks and benefits. To what extent do you agree or disagree with the following statements?<br>- There may be negative impacts of carbon dioxide removal technologies on the environment<br>- Carbon dioxide removal technologies will lower the drive to cut carbon emissions<br>- Carbon dioxide removal technologies are being driven more by profit than by the public interest<br>- Carbon dioxide removal technologies will mainly benefit rich countries and impact on poor countries<br>- Carbon dioxide removal technologies could help to provide more time to reduce emissions<br>- It will be cheaper to use carbon dioxide removal technologies than to reduce the consumption of fossil fuels<br>- Carbon dioxide removal technologies will help slow climate change down faster than by simply cutting greenhouse gas emissions<br>- Carbon dioxide removal only deals with the symptoms and not the causes of emissions	Strongly agree, Tend to agree, Neither agree nor disagree, Tend to disagree, Strongly disagree
Participants were then given a piece of information to read about enhanced weathering (shown in Figure 1) before being asked the following items.	
Q6. Please tell us what thoughts or images came to mind when reding this information about enhanced weathering?	(open-ended)

Q7. Before today, how much if anything, would you say that you know about enhanced weathering?	- I know a great deal about enhanced weathering<br>- I know a fair amount about enhanced<br>- I know just a little about enhanced weathering<br>- I have heard of enhanced weathering but know almost nothing about it<br>- I have not heard of enhanced weathering before today
<u>Q8. Risks:</u><br>- Mining and crushing minerals for enhanced weathering could cause environmental damage<br>- Mining, crushing and transporting minerals for enhanced weathering could result in high energy costs<br>- We don't know enough about the long-term effects of enhanced weathering<br>- There may be impacts on plants and animals in the marine environment<br><u>Benefits:</u><br>- Enhanced weathering could improve growing conditions for crops<br>- Enhanced weathering may help reduce the impact of climate change on the oceans<br>- Enhanced weathering could benefit the environment as minerals may improve crop resistance to pests, reducing pesticide use	Strongly agree, Tend to agree, Neither agree nor disagree, Tend to disagree, Strongly disagree
Q9. In general, how do you feel about enhanced weathering?	Very negatively, Negatively, Neither negatively or positively, Positively, Very positively
Q10.<br>- Enhanced weathering is contrary to nature<br>- Enhanced weathering disturbs the order of nature<br>- Trying to influence the climate system by enhanced weathering reflects human arrogance<br>- Human's goal to change the climate system by enhanced weathering is immoral	Strongly agree, Somewhat agree, Neither agree or disagree, Somewhat disagree, Strongly disagree
Q11. Overall, to what extent would you support enhanced weathering to tackle climate change?	Strongly support, Tend to support, Neither support or oppose, Tend to oppose, Strongly oppose
Q12. Would you support the use of enhanced weathering as a way to reduce carbon emissions?	Not at all, Probably not, Not sure, Probably yes, Definitely
Q13. My country's resources (finances, energy and land) should be used to implement enhanced weathering	Strongly agree, Tend to agree, Neither agree nor disagree, Tend to disagree, Strongly disagree
Q14. Please explain your choice regarding whether or not you would support the use of enhanced weathering.	(open-ended)

ESM3: Open ended coding frameworks

Association EW complete framework

Categories	Australia %	UK %	US %	Summary Description
**Positive**	14.5	13.8	13.5	All positive words, emotions, expressions that do not fit into any other category
**Impacts on environment and wildlife**	11.8	12.7	14.1	All mentions of damage to the environment and organisms, waterways, lands
**Risky**	10.7	11.4	9.7	Risks outweigh benefits, bandaid, temporary solution, causing more damage
**Misc**	10.2	9.3	10.5	Nonsense responses, unrelated, all others that do not fit
**None/Don't know**	10	13.5	10	Don't know, not sure, none
**Negative**	9	10.2	9.6	All negative words, emotions, expressions that do not fit into any other category
**Potentiality**	6	5.5	5.4	Mention if it will work or not, whether worthwhile or necessary
**Confused/Complex**	5.6	6.4	3.7	Mentions complex process, technical or complicated more generally, confused, hard to understand
**Mining**	4.6	2.1	3.8	Reference to mining, industrial process (machinery, equipment), materials, resource intensive, minerals etc
**Costs**	4.1	4.3	5.8	Finance, cost, business, greed, reference to resources (not minerals/related to mining)
**Sceptical**	3.2	1.6	2	Climate change deniers, scaremongering, waste of time, unlikely
**Pollution**	2.9	2.4	2.9	General mentions of pollution that are not tied to human or environmental impacts
**Natural**	2.8	1.6	1.8	Natural process/products, natural cycle, unnatural or artificial, playing God
**Climate change**	1.5	1.9	2.2	Mentions of climate change, general carbon dioxide that does not seem sceptical
**Farms**	1.3	1.2	1.8	Farm machinery (not related to mining/minerals), crops, food production
**Human caused**	1	1.3	1.3	Any mention of human involvement
**Impacts on humans and health**	0.4	0.7	1.7	Any mention of damage to humans or to our food/water supplies
**Responsibility**	0.4	0.1	0.3	Queries of who is responsible, governments

Q6. Please tell us what thoughts or images came to mind when reading this information about enhanced weathering?

Support for EW complete framework

Categories	Summary Description
**None/Don't know**	Left blank, none, can't think of any, need to know more
**Climate change**	Any reference to climate change or global warming that is not sceptical or mention of carbon dioxide
**Impact environment**	All responses that mention damage to environment, animals and wildlife and concern about environmental damage
**Impact humans**	Mentions of life or humans being affected negatively
**Positive for environment**	Comments around the environment improving or benefiting the planet
**Positive for humans**	Responses that mention positive impacts for humans, society or people, rare code
**Positive**	Comments that are positive and do not fit into other categories
**Negative**	Comments that cannot be coded elsewhere and are generally negative
**Bandaid/temporary**	Responses that talk about it being a bandaid, temporary fix
**Cost**	All mentions of money, resources and cost that would be needed
**Naturalness**	Mention of nature or it being natural or unnatural
**Sceptical**	Responses that seem sceptical or denialist
**Misc**	Responses that do not make sense, reword question, yes, no, do not answer question
**Risks**	Any mention of it being risky or harmful or not knowing about damage yet or being more negative than positive
**Benefits**	Responses that mention it being beneficial or of benefit or outweighing the risk
**Risks vs benefits**	All responses that seem unsure about cost/benefits of process or a desire to see further information to make a decision based on risks/benefits
**Research/evidence**	Any mention of research, evidence, need for trials or testing, it not being proven or having proof
**Alternatives**	Mentions of the need for other solutions, changing energy, other actions that people/society takes

Q14. Please explain your choice regarding whether or not you would support the use of enhanced weathering.

ESM4 Descriptive statistics

Country:	AUS	UK	US
**Variable**	**M (SD)**	**M (SD)**	**M (SD)**
Concern climate change	2.97 (0.92)	3.15 (0.78)	3.01 (0.94)
Worry climate change	3.21 (1.18)	3.27 (1.06)	3.20 (1.23)
Science/Tech solve climate change	3.73 (1.20)	3.84 (1.17)	3.85 (1.24)
Prior knowledge	1.45 (0.85)	1.53 (0.92)	1.71 (1.07)
Risks EW	4.03 (0.70)	3.95 (0.65)	3.95 (0.75)
Benefits EW	3.31 (0.76)	3.35 (0.67)	3033 (0.77)
Affect	2.92 (0.89)	2.97 (0.81)	2.97 (0.93)
Messing with nature	3.38 (0.88)	3.40 (0.75)	3.30 (0.88)
Support use EW to reduce carbon emissions	3.12 (0.97)	3.20 (0.86)	3.11 (0.99)
Support EW CC	3.04 (0.97)	3.17 (0.90)	3.12 (1.02)
Support EW Resources	3.04 (1.01)	3.12 (0.93)	3.01 (1.01)
Support use EW (scale)	3.07 (0.87)	3.16 (0.77)	3.08 (0.90)

Means and SDs for each variable included across Australia, the UK, and the US
